# The Treatment of Sterility

**Published:** 1886-07

**Authors:** 


### The Treatment of Sterility.

The most common cause, according to Hewitt, is a conoidal shape of the cervix. Generally, the vaginal portion of the neck is elongated, and the whole cervical canal is bent upon itself. The uterine orifice looks forward; therefore, conception is difficult.

Two curative methods are recommended: first, amputation of a portion of the neck; second, division of the cervix by median incision through the posterior wall. A patient having a cervix very much flexed and elongated, suffering with difficult micturition and painful locomotion on account of anteflexion, married four years, and having no children, presented herself to Hewitt, who performed the following operation: After drawing down the neck, he excised upon the median line of the posterior surface, a vertical portion of mucous membrane an inch long and half an inch wide. Then he stitched the edges above and below. The effect of this operation was to shorten the cervix on its posterior wall, to draw the os backward, and to straighten the vaginal portion of cervix.

When three sutures were inserted, the os, which previously looked forward, pointed directly downward. After several days the sutures were removed. The patient subsequently became pregnant.

The author prefers this operation to amputation of the vaginal portion, because it preserves the natural os and leaves the cervical canal intact. There is also less mutilation than by that of Emmett's.—*Journal de Medecine*, April 4, 1886.

